# The United States Senate and the Tale of Two Disasters

**Published:** 1912-05

**Authors:** C. A. L. Reed


### The United States Senate and the Tale of Two Disasters.

The Titanic went down with sixteen hundred souls on board. Its loss represented millions upon millions of wealth. The world now knows that it was a case of recklessness, a sacrifice to preventable causes. It was the greatest single marine disaster in history. The world was shocked. Rulers telegraphed words of condolence to each other. The United States Senate properly and promptly appointed an investigation committee that hastened with all speed to beat the survivors to port. Senatorial brows were thoughtful and senatorial pens were busy to formulate legislation designed to prevent recurrrence of the calamity. Senator Raynor and others anathematized the steamship company and its managers. Others did the same. A common human impulse has ratified the celerity and purpose of all this present activity at Washington.

But suppose that during the last twelve months not only the Titanic, but four hundred Titanics, each carrying 1500 persons, had gone to the bottom of the sea. Or suppose that every craft of every class belonging to the United States Navy, with every officer and man aboard, had been lost during the last year. Then suppose that that accident had happened, not once but ten times over during that same period. Suppose that all that great loss—either four hundred Titanics or ten United States navies—had been the result of entirely preventable causes. Is it conceivable that under such circumstances Senator Raynor and other Senators would have arisen in their places to make some observation on the occurrence? Would a committee on investigation have been appointed, a bill drafted and legislation enacted in something less than four years? Yet Senator Raynor and his colleague have been guilty of precisely such indifference and such negligence in the presence of an annual disaster that, in loss of life and treasure, is the equivalent of the loss of four hundred Titanics or ten United States navies.

They can not plead ignorance of the facts. By their own act, by the act of Congress, a bureau of government was created that has furnished information to both branches of that body to the effect that over 600,000 die every year from preventable diseases. Statisticians and economists estimate the monetary loss thus incurred at more than two billion dollars annually. The whole disaster, including deaths and loss of money, is due to somebody's carelessness. The United States government is not doing its duty in the premises. The Owen bill, to rectify this situation, was introduced in the Senate four years ago. Senator Raynor and his colleagues have permitted it to slumber in committee during all of that time, while, so far as the loss of life and treasure were concerned, Titanics have been going down at the rate of more than one a day. And now, within the last few weeks, when a play is to be made to catch every possible vote at the coming election, the committee of the Senate insults the intelligence and humanity of the country by reporting out a puny and emasculated measure.

The Committee on Public Health and National Quarantine, that has been responsible for this disgraceful delay and that is now responsible for this equally disgraceful report, consists of Senators Thomas S. Martin, Virginia, Chairman; Samuel D. McEnery, Louisiana; Charles A. Culberson, Texas; Duncan N. Fletcher, Florida; Henry A. Dupont, Delaware; Jonathan Bourne, Jr., Oregon; Reed Smoot, Utah; Joseph L. Bristow, Kansas; Coe L. Crawford, South Dakota. The names are given because the time has come when men guilty of such derelictions are to be held politically responsible to their constituents, as they ought to be held personally responsible to the sorrowing but bereaved friends of their victims. We ask every physician in Virginia, Louisiana, Texas, Florida, Delaware, Utah, Kansas and South Dakota to bring these facts and their significance to the attention of his patrons, and especially the attention of every person who directly or indirectly has been victimized by preventable diseases. The physicians and electors of Oregon have attended to the matter of Jonathan Bourne, Jr., whom they have elected to stay at home.

Senator Raynor is right. Investigate the steamship company and its officials for criminal carelessness. The people, too, are right. Investigate Senator Raynor and his colleagues. Investigate him and his colleagues for equally culpable neglect in the presence of even greater disaster. Let there be no doubt but that the people will do this very thing, and do it promptly.—C. A. L. Reed, in *Lancet-Clinic,* April 27th.

